# Accuracy of an AI-based automated plate reading mobile application for the identification of clinical mastitis-causing pathogens in chromogenic culture media

**DOI:** 10.1038/s41598-023-50296-w

**Published:** 2024-01-12

**Authors:** Breno Luis Nery Garcia, Cristian Marlon de Magalhães Rodrigues Martins, Lucas Faria Porto, Diego Borin Nobrega, Marcos Veiga dos Santos

**Affiliations:** 1 Department of Animal Nutrition and Production, School of Veterinary Medicine and Animal Science, University of São Paulo, Pirassununga, São Paulo 13635-900 Brazil; 2 Rúmina S.A., Belo Horizonte, Minas Gerais 30320-130 Brazil; 3 Faculty of Veterinary Medicine, University of Calgary, Calgary, AB T2N 1N4 Canada

**Keywords:** Antimicrobials, Bacteria, Computational biology and bioinformatics, Image processing, Machine learning

## Abstract

Using on-farm microbiological culture (OFC), based on chromogenic culture media, enables the identification of mastitis causing pathogens in about 24 h, allows rapid decision making on selective treatment and control management measures of cows with clinical mastitis (CM). However, accurate interpretation of OFC results requires trained and experienced operators, which could be a limitation for the use of OFC in dairy farms. Our hypothesis was that AI-based automated plate reading mobile application can analyze images of microorganisms’ colonies in chromogenic culture media with similar diagnostic performance as a trained specialist evaluator. Therefore, the aim of the present study was to evaluate the diagnostic accuracy of an AI-based application (Rumi; OnFarm, Piracicaba, São Paulo, Brazil) for interpreting images of mastitis causing microorganism colonies grown in chromogenic culture media. For this study two trials were organized to compare the results obtained using an AI-based application Rumi with the interpretation of: (1) a trained specialist, using MALDI-TOF MS as the gold standard; (2) farm personnel users (FPU). In trial 1, a total of 476 CM milk samples, from 11 farms located in São Paulo (n = 7) and Minas Gerais (n = 4), southeast Brazil, were inoculated in chromogenic culture media plates (Smartcolor 2, OnFarm, Piracicaba, São Paulo, Brazil) by specialists under lab conditions, and digital images were recorded 24 h after incubation at 37 °C. After that, all the 476 digital images were analyzed by the Rumi and by another specialist (who only had access to the digital images) and the diagnostic accuracy indicators sensitivity (Se) and specificity (Sp) were calculated using MALDI-TOF MS microbiological identification of the isolates as the reference. In Trial 2, a total of 208 CM milk samples, from 150 farms from Brazil, were inoculated in chromogenic culture media plates by FPU, and the results of microbiological growth were visually interpreted by FPU under on-farm conditions. After visual interpretation, results were recorded using an OnFarmApp application (herd manage application for mastitis by OnFarm, Piracicaba, São Paulo, Brazil), and the images of the chromogenic culture plates were captured by the OnFarmApp to be evaluated by Rumi and Bayesian Latent Class Models were performed to compare Rumi and the FPU. In Trial 1, Rumi presented high and intermediate accuracy results, with the only exception of the low *Enterococcus* spp.’s Se. In comparison with the specialist, Rumi performed similarly in Se and Sp for most groups of pathogens, with the only exception of non-aureus staphylococci where Se results were lower. Both Rumi and the specialist achieved Sp results > 0.96. In Trial 2, Rumi had similar results as the FPU in the Bayesian Latent Class Model analysis. In conclusion, the use of the AI-based automated plate reading mobile application can be an alternative for visual interpretation of OFC results, simplifying the procedures for selective treatment decisions for CM based on OFC.

## Introduction

Mastitis is one of the main reasons for the use of antimicrobials on dairy farms, representing around 80% of the total use of antimicrobials in dairy production^1^. However, antimicrobial treatment of all clinical mastitis (CM) cases is not always justified, because around 40% of CM cases have no isolation of mastitis-causing pathogens. Moreover, among culture-positive results, some pathogens respond poorly to antimicrobial therapy, and/or have a high rate of spontaneous cure (e.g., *Escherichia coli*^2^). The rapid and accurate diagnosis of mastitis pathogens is an important element of an effective protocol for selective therapy of clinical mastitis^3^. Different on-farm microbiological culture (OFC) methods have been used for rapid on-farm identification of mastitis pathogens, including the use of chromogenic culture media^4^. The adoption of OFC enables selective treatment for CM, which can reduce antimicrobial use by 50%^5,6^, without reduction on bacteriological cure risk^7^.

Interpreting OFC results requires adequate training and experienced farm personnel, which can be a limitation to the adoption of OFC systems in dairy herds. Substantial differences in accuracy are observed between specialists and untrained users, showing that specific training is critical to appropriate mastitis treatment decisions based on OFC results^6^. Furthermore, in chromogenic media-based OFC, because of the subjectivity of color interpretation of colonies, variation in diagnostic performance is observed between specialists and farm personnel users (FPU)^8^, which can compromise the diagnostic performance of the method.

Automation in the culture media evaluation can be an alternative to minimize subjectivity in the OFC results interpretation. It has been reported that automatic evaluation systems can present similar accuracy as the evaluation of a trained specialist when using urine samples^9^. Automating procedures and diagnoses using computational techniques is not a recent subject in research. Solutions using machine learning for automatic image diagnosis have been explored with several applications, such as: analysis using X-ray images^10^, photo-anthropometric analysis of facial images^11,12^, identification of cancer cells^13^, dental sexual dimorphism classification using radiography images^14^, classification of bacteria and nematodes on microscope images^15,16^. The automation of the chromogenic culture results interpretation comprises the use of artificial intelligence (AI) to analyze images of culture media plates and, in real time, categorize them as positive or negative for specific pathogens based on interpreting the color and colony characteristics of specific microorganisms^17^.

AI-based application has been tested in human medicine and have achieved satisfactory accuracy (> 80% sensitivity) in interpreting microbiological culture results in urinary tract isolates^9,18^; screening for methicillin-resistant/sensible *Staphylococcus aureus* infection in patients in intensive care units^17^; detection of group B *Streptococcus* in women^19^ and *Streptococcus pyogenes* isolates in pharyngitis cases^20^. However, there are no studies evaluating AI-based application method for chromogenic culture media used for mastitis-causing pathogens identification.

We hypothesize that employing an AI-driven automated mobile application for plate reading, designed to interpret images of prevalent mastitis-causing bacteria in chromogenic culture media, can achieve diagnostic accuracy comparable to that of a trained specialist. Such a technological advancement holds the potential to streamline milk culturing on farms, mitigating the risk of diagnostic errors that may arise when untrained personnel are responsible for plate reading. Therefore, the aim of the present study was to evaluate the diagnostic accuracy of an AI-based application (Rumi; OnFarm, Piracicaba, São Paulo, Brazil) for interpreting images of mastitis-causing microorganism colonies grown in chromogenic culture media. The study was organized into two trials with the following objectives: 1) Assess the diagnostic accuracy of Rumi in contrast to a trained specialist, using MALDI-TOF-MS as the gold standard; 2) compare the accuracy of Rumi and FPU to read plates on farms, which will serve as a proxy to estimate the improvements in diagnostic accuracy attributed to the implementation of Rumi (Table 5).

## Results

### Trial 1

Nearly half of the CM samples (267/476) were considered negative, while 43.9% (209/476) were considered positive. From all samples, 20.4% (97/476) contained isolates with two distinct morphologies (mixed culture). In total, 306 isolates from 46 different species were identified in MALDI-TOF MS. Among the groups of pathogens that can be identified by Smartcolor2, *Lactococcus* spp., *Pseudomonas* spp., yeast, *Prototheca* spp., other Gram-negative and other Gram-positive microorganism had a low frequency of isolation (n < 10) and were, therefore, grouped as “other pathogens”. The most frequently isolated pathogen group was non-*aureus* staphylococci (10.9%) followed by *E. coli* (7.6%) and *Staphylococcus aureus* (7.3%; Table 1).

**Table 1:** Frequency isolation of mastitis-causing pathogens from 476 clinical mastitis samples cultured in blood agar and identified by MALDI-TOF MS.

Variable	Frequency (n)	%
Total	476	100.0
Negative	267	56.1
Mixed culture	97	20.4
Gram-positive	214	45.0
*Streptococcus agalactiae*	9	1.9
*Streptococcus dysgalactiae*	13	2.7
*Streptococcus uberis*	19	4.0
*Enterococcus* spp.	10	2.1
*Lactococcus* spp.	6	1.3
*Staphylococcus aureus*	35	7.3
Non-*aureus* staphylococci	52	10.9
Other Gram-positive pathogens	70	14.7
Gram-negative	92	19.3
*Escherichia coli*	36	7.6
*Klebsiella* spp.	20	4.2
*Enterobacter* spp.	5	1.0
*Serratia* spp.	5	1.0
*Pseudomonas* spp.	8	1.7
Yeast and *Prototheca* spp.	10	2.1
Other Gram-negative pathogens	8	1.7

Both the specialist and Rumi had Sp results > 0.96 for all the groups of pathogens evaluated (Table 1). The specialist Se ranged from 0.60 (*Enterococcus* spp.) to 0.97 (*E. coli*), while Rumi’s Se ranged from 0.20 (*Enterococcus* spp.) to 0.97 (*Klebsiella* spp./*Enterobacter* spp./*Serratia* spp.). There were no significant differences in the Se and Sp of Rumi and the specialist to identify most group of pathogens evaluated (Table 2). For non-aureus staphylococci, Rumi had lower Se (0.94) than the specialist (0.73).A list of cross tabulated results according to the MALDI-TOF MS status is available as supplemental material (Tables S1).

**Table 2:** Diagnostic sensitivity (Se) and specificity (Sp) of the visual identification of mastitis-causing pathogens from clinical mastitis samples (n = 476) in chromogenic culture media triplates (SmartColor 2—OnFarm. Brazil) made by a trained specialist and by an artificial intelligence-based application (Rumi; OnFarm. Piracicaba. São Paulo. Brazil).

	Frequency (n)	Se (CI)		Sp (CI)	
*Streptococcus agalactiae/Streptococcus dysgalactiae*	25				
Specialist		0.86	(0.65–0.97)	0.97	(0.95 -0.98)
Rumi		0.68	(0.45–0.86)	0.98	(0.96–0.99)
*Streptococcus uberis*	24				
Specialist		0.89	(0.67–0.99)	0.98	(0.96–0.99)
Rumi		0.84	(0.60–0.97)	0.96	(0.94–0.97)
*Enterococcus* spp.	13				
Specialist		0.60	(0.26–0.88)	0.97	(0.95–0.98)
Rumi		0.20	(0.02–0.56)	0.97	(0.95–0.99)
Other Pathogens	7				
Specialist		0.61	(0.49–0.71)	0.98	(0.96–0.99)
Rumi		0.38	(0.29–0.49)	0.98	(0.97–0.99)
*Klebsiella* spp./*Enterobacter* spp./*Serratia* spp.	31				
Specialist		0.93	(0.78–0.99)	1.00	(0.98–1.00)
Rumi		0.97	(0.83–1.00)	0.99	(0.98–1.00)
*Escherichia coli*	47				
Specialist		0.97	(0.88–1.00)	0.99	(0.98–1.00)
Rumi		0.89	(0.74–0.97)	0.99	(0.98–1.00)
*Staphylococcus aureus*	35				
Specialist		0.83	(0.66–0.93)	0.99	(0.98–1.00)
Rumi		0.74	(0.57–0.87)	0.98	(0.97–0.99)
Non-*aureus* staphylococci	70				
Specialist		0.94^a^	(0.84–0.99)	0.99	(0.98–1.00)
Rumi		0.73^b^	(0.61–0.84)	0.98	(0.97–1.00)

The microbiological identification using MALDI-TOF MS was considered the gold standard. CI = 95% confidence intervals.

^a,b^Accuracy parameters followed by different superscripts for the same pathogen category denote statistically significant differences at 5% significance level.

### Trial 2

According to our case definition, 28.8% (60/208) of the samples were considered negative, while 71.1% (148/208) were positive. A total of 31.2% (65/208) of samples had mixed cultures. *Lactococcus* spp., *Serratia* spp., *Pseudomonas* spp., yeast, *Prototheca* spp., other Gram-negative and other Gram-positive microorganism had a low frequency of isolation (n < 10) and were not considered for Bayesian Latent Class models. Non-aureus staphylococci was the most frequently isolated group (35.1%) followed by *Streptococcus agalactiae/dysgalactiae* (13.0%) and *Streptococcus uberis* (11.5%; Table 3).

**Table 3:** Distribution of mastitis-causing pathogens of 208 clinical mastitis samples cultured on chromogenic culture media triplate (Smartcolor 2. OnFarm. Piracicaba. São Paulo. Brazil).

Variable	Frequency (n)	%
Total	208	100.0
Negative	60	28.8
Mixed culture	65	31.2
*Streptococcus agalactiae*/*Streptococcus dysgalactiae*	27	13.0
*Streptococcus uberis*	24	11.5
*Enterococcus* spp.	16	7.7
*Staphylococcus aureus*	16	7.7
Non-*aureus* staphylococci	73	35.1
*Escherichia coli*	18	8.6
*Klebsiella* spp./*Enterobacter* spp./*Serratia* spp.	10	4.8
Other pathogens	55	26.4

In total, 35 models (5 models per pathogen group) were run. In general, Rumi performed as well as the FPU for all groups of pathogens evaluated (Table 2). No statistically significant differences in Se and Sp were observed between Rumi and FPUs for identifying isolates, irrespective of bacterial species (Table 4). These comparisons were not affected by our choice of prevalence or diagnostic prior information, as demonstrated by sensitivity analysis (Tables S3, S4). A list of cross tabulated results for the two diagnostic procedures is available as supplemental material (Tables S2).

**Table 4:** Mode Sensitivity (Se) and Specificity (Sp) of the visual identification of mastitis-causing pathogens from clinical mastitis milk samples (n = 208) in chromogenic culture media triplates (SmartColor 2—OnFarm. Brazil) made by farm personnel users (FPU) and by an artificial intelligence-based application (Rumi; OnFarm. Piracicaba. São Paulo. Brazil).

	Comparison	Mode
*Streptococcus agalactiae/Streptococcus dysgalactiae*	Se Rumi > Se FPU	0.06
	Sp Rumi > Sp FPU	0.67
*Sreptococcus uberis*	Se Rumi > Se FPU	0.37
	Sp Rumi > Sp FPU	0.72
*Enterococcus* spp.	Se Rumi > Se FPU	0.33
	Sp Rumi > Sp FPU	0.33
*Klebsiella* spp./*Enterobacter* spp./*Serratia* spp.	Se Rumi > Se FPU	0.5
	Sp Rumi > Sp FPU	0.65
*Escherichia coli*	Se Rumi > Se FPU	0.41
	Sp Rumi > Sp FPU	0.59
*Staphylococcus aureus*	Se Rumi > Se FPU	0.18
	Sp Rumi > Sp FPU	0.92
Non-aureus staphylococci	Se Rumi > Se FPU	0.22
	Sp Rumi > Sp FPU	0.69

## Discussion

The diagnostic accuracy of the OFC depends on various factors, which includes the pathogen and culture media diagnostic accuracy, the level of training and experience of the operator in interpreting the results^8^. AI-based application that automatically interprets OFC results can be an alternative to enhance the diagnostic accuracy of OFC systems. The present study was divided in two trials which aimed to: (a) evaluated the performance of an AI-based application for microbiological diagnosis of mastitis-causing pathogens using chromogenic culture media images (b) evaluate if this AI-based application can improve the accuracy OFC diagnosis of CM in comparison to a farm personal user.

### Trial 1

Rumi demonstrated high Se for the most prevalent environmental pathogens evaluated (*Streptococcus uberis; Klebsiella* spp./*Enterobacter* spp*.*/*Serratia* spp. and *Escherichia coli*), indicating that, for this group of pathogens, the AI-based application can perform comparably to a specialist in interpretating chromogenic culture media results. Considering the majority of the herds using the OFC system were compost-barn farms, and that environmental streptococci and *Escherichia coli* were the most prevalent causes of CM in compost-barn Brazilian herds^21^, achieving a high diagnostic accuracy for these pathogens is crucial for implementing adequate CM treatments. Additionally, for the Gram-negative pathogens evaluated (*Klebsiella* spp./*Enterobacter* spp*.*/*Serratia* spp. and *Escherichia coli*) both the specialist and Rumi had all high diagnostic accuracy results, indicating the identification capacity of the chromogenic culture media for those groups, as observed by Granja et al.^4^ and Ferreira et al.^22^. Moreover, the results indicate that Rumi was able to differentiate between the two groups, *Klebsiella* spp./*Enterobacter* spp./*Serratia* spp. and *Escherichia coli*. This ability is critical for decision-making on mastitis treatment using OFC results, since *Escherichia coli* usually does not require antibiotic treatment because of its high spontaneous cure rate^2^, while *Klebsiella* spp. would benefit of antimicrobial therapy in the treatment of non-severe and clinical mastitis^23^.

Regarding the predominantly contagious pathogens evaluated, although Rumi’s Sp results were all > 0.95, the Se results were < 0.80. As contagious pathogens control is predominantly related to prevention of transmission and identification of the positive cows^24^, Se results are the most important accuracy predictors. Rumi’s Se results for *Staphylococcus aureus* was 0.73, which can be related to the misidentification between *Staphylococcus aureus* and non-aureus staphylococci, since 5 out of 9 FN results were classified as non-aureus staphylococci. However, despite of been numerically greater, the specialist Se results for *Staphylococcus aureus* and the *Streptococcus agalactiae/Streptococcus dysgalactiae* group weren’t statistically different at a 5% significance level, which denotes that the low Se is probably related to the accuracy of the chromogenic culture media itself for those species. Similar results were reported by Garcia et al.^25^ using the same chromogenic culture media, but with post-partum subclinical mastitis samples (Se = 0.67), and those results were attributed to inconsistencies in the colony color pattern of this pathogen, in the chromogenic culture media. *Staphylococcus aureus* identification is particularly important because of its high transmission capacity, low cure rates and high resistance to antimicrobial treatment^24,26^. However, *Staphylococcus aureus* is mostly associated with subclinical mastitis, and the primarily focus of Rumi’s microbiological identification is related to CM cases for selective treatment decisions.

The group non-*aureus* staphylococci was the only one in which a statistical difference in accuracy parameter results was observed between the specialist and Rumi. A lower Se was observed for Rumi compared to the specialist, potentially resulting in fewer treatment of positive cows for non-*aureus* staphylococci, since FN results correspond to positive samples classified as negative within this group. This difference can possibly be atributed to the chromogenic culture media not having a specific color definition for non-*aureus* staphylococci as a group, and classifying it as any color other than pink. Rumi’s supervised machine-learning model was developed based on digital images of SmartColor 2 plates labeled with the presumptive microbiological identification result. Considering that the non-aureus staphylococci group presents about 11 different species causing mastitis^27^, Rumi was exposed to a broad variation in colony color and morphology in the training process, which may have decreased the accuracy of the identification for the group.

Although a difference was observed in the Se results of one of the pathogen groups evaluated, Rumi performed similarly as the specialist in Sp results. Both the specialist and Rumi presented satisfactory results regarding Sp, for all groups of pathogens evaluated. Considering that the selective treatment of CM presupposes that only cases which will benefit from treatment are treated^3^, a high Sp is essential for adopting OFC results as a criterion for therapy. For most of the evaluated groups of pathogens (with the only exception of *Escherichia coli*), an FP result could lead to unnecessary treatment, and so a high Sp is crucial for the reduction of antimicrobial therapy on mastitis control, which is one of the primary reasons for the OFC implementation on dairy herds.

The only group of pathogens witch Rumi presented diagnostic accuracy results <0.60 was *Enterococcus* spp. (Se = 0.20). Although, Rumi’s low accuracy for this group can be probably attributed to the chromogenic culture media performance itself, since both the specialist and Rumi evaluation presented low Se. The specialist interpretation had 4 FN results, while Rumi had 8 FN results for *Enterococcus* spp. identification. Half of Rumi’s FN results (4 out of 8) were in the same samples that were classified as FN for the specialist’s evaluation, indicating that this incorrect identification is associated with the chromogenic culture media performance. Those results agree with the diagnostic accuracy obtained by Granja et al. (2021), evaluating the same chromogenic culture media for clinical mastitis samples (Se = 0.43) and subclinical mastitis (Se = 0.25). Additionally, *Enterococcus* spp. and *Streptococcus* spp. genus has narrow phenotypic similarity which leads to a difficult morphological differentiation^28^, even in chromogenic media, in which the identification is made by the color patterns. Ferreira et al.^22^ found, in Accumast chromogenic culture media, the group *Lactococcus*/*Enterococcus* as the most common cause of FP results, leading to a low PPV (0,538±0.26), as it was observed in our study. Probably, the low isolation frequency of *Enterococcus* spp. (n = 13) has compromised the results of Se, both for the specialist and Rumi evaluation. Considering that the criteria for separately calculating the diagnostic accuracy predictors (out of the “other pathogens” group) were, at least 10 isolations, *Enterococcus* spp. had only 3 isolates above the breakpoint.

It is necessary to consider that, our gold standard method used blood agar as the primary isolation medium incubated by 48-hour period (with inspections at 24 and 48 hours), while the SmartColor 2 plates had a period incubation of only 24 hours. This difference of incubation period between the methods can be considered a limitation of the study because some pathogens have fastidious growth and demand a longer period of incubation (e.g., 2 to 3 days for growth of *Corynebacterium* spp.^29^). Although, 24 hours is the maximum safe awaiting period recommended for decision-making on CM treatment without affecting cure rates^3,30^. In this sense, prolonging the incubation period of SmartColor 2 plates to match the gold standard methodology would bias our results, once this procedure is not replicable on the field. Another potential limitation is the use of swabs instead of platinum loops for the inoculation procedures of both methodologies, which, due to the lack of a standardized volume, could increase the risk of FP results. However, as the OFC procedure is based on the swab inoculation, using a platinum loop only on blood agar would lead to additional bias, as low colony-forming unit samples could be erroneously considered FP on SmartColor 2 due to the inoculation volume difference between methods. To mitigate bias, we chose to apply the same procedure for both, SmartColor2 and blood agar.

### Trial 2

The results of the Bayesian Latent Class Model indicate no differences in accuracy parameters between FPU and Rumi. Although no accuracy improvement was found, the use of Rumi can help in simplifying the OFC identification process, reducing the need for an additional operation. Considering that, currently, all FPU need to be trained by OnFarm employees to perform microbiological identification, the use of Rumi can make the process simpler and faster for new FPU that are implementing the selective treatment of CM based on OFC. This simpler implementation process can also be important for farms to decide on implementing the OFC system, as changes in management can present challenges. Additionally, as Rumi automatically provides the microbiological identification, there is no need for manually registering the results on the CM management recordings, which improve the record-keeping efficiency.

Despite the lower Se results of Rumi in comparison to the specialist in Trial 1, no difference was observed in the diagnostic accuracy parameters of the non-*aureus* staphylococci identification between Rumi and FPU. This denotes that there is no disadvantage in using Rumi for microbiological identification of this group. Although, concurrently, it indicates that the OFC method itself does not have a good diagnostic performance for this group of pathogens, since the use of Rumi did not affect the accuracy, even though it was lower than the specialists Se in Trial 1.

It should be pointed out that the Rumi and FPU evaluations were not performed under the same conditions. The FPU had the advantage of holding and moving the chromogenic culture media Petri dishes for the interpretation of the microbiological colonies, while Rumi only had access to the digital image of each plate. Nevertheless, similar accuracy parameter results were observed for all groups of pathogens evaluated. Even though, none of Rumi’s evaluations presented lower diagnostic accuracy results than an FPU, the use of Rumi did not improve the diagnostic accuracy of the OFC method as it was hypothesized. Our results indicated that there is still room for improvement in the development of the AI-based application for chromogenic culture media plate reading. Using a greater number of images for the training could be an alternative, especially for some groups of pathogens with low frequencies of isolation (e.g. *Enterococcus* spp.) or a broad difference in colony morphology among the species within a specific group (e.g. non-*aureus* staphylococci). Additionally, it is necessary to highlight that the frequency of isolation found for some groups of pathogens in Trial 2 limited the power of the test, which can address the sample size as a limitation of the trial.

## Methods

### On-farm microbiological culture system

**Figure 1:**
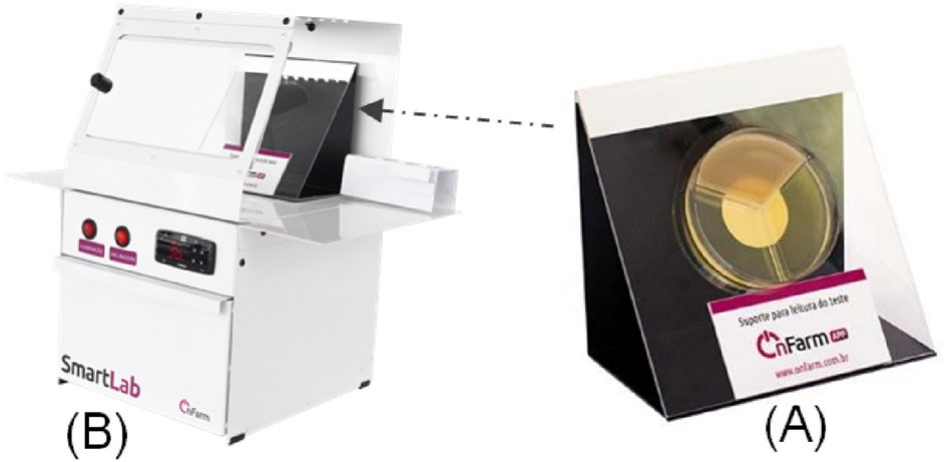
Reader Support (**A**) located at the SmartLab Incubator (**B**; OnFarm, Piracicaba, São Paulo, Brazil) used to take pictures for model training and performance evaluation. *Source*: OnFarm (Piracicaba, São Paulo, Brazil).

The OFC system provided by OnFarm (Piracicaba, São Paulo, Brazil) is currently used by approximately 2,000 dairy farms located in 20 different states of Brazil. The OFC system is composed of the following items: (1) triplate chromogenic culture media plates (SmartColor 2), (2) incubators equipped with a Petri dish reader support, a dark background (for photography of SmartColor 2 plate) and luminosity with 6000 K LED light (SmartLab; Fig. 1) and (3) a mobile application for herd and cow mastitis data recording (OnFarmApp). When implementing OnFarm’s system, the farms receive an online training by OnFarm’s team about how to operate the OnFarmApp and how to perform OFC.

OFC procedures are generally carried out by trained employees, chosen from the farm existing staff, following the farm's own criteria, but with no specific qualification required at first (only the initial training by OnFarm). All CM cases are identified by farm personnel, based on the identification of abnormalities in milk secretion or in the udder of cows. The OnFarm training program emphasizes protocols for obtaining milk samples aseptically, inoculating, and incubating culture plates, and utilizing the OnFarmApp for storing information and reading culture plates. After the microbiological identification, FPU are oriented to record pictures of the SmartColor 2 triplates on the reader support of the SmartLab and upload it in the OnFarmApp along with the CM case data. The images are recorded by individuals with no advanced technical knowledge of photography using a variety of phone devices, and stored at a resolution of 2500 × 2500 pixels, 24 bits of color. When FPU have doubts regarding the identification of specific pathogens, a remote inspection of uploaded images can be requested.

### Chromogenic culture media interpretation

The Smartcolor 2 triplate, whose images were used in the study, comprises of a triplate Petri dish composed of three different selective chromogenic culture media: Section 1: *Streptococcus* spp.; Section 2: *Staphylococcus aureus* and *Staphylococcus* spp. and Section 3: gram-negative bacteria. Interpretation of the growth in each section of the plate was done according to the following colony colors:

- Section 1—(a) dark blue = *Streptococcus uberis*; (b) turquoise blue = *Streptococcus agalactiae* or *Streptococcus dysgalactiae*; (c) purple = *Enterococcus* spp.; (d) lilac = *Lactococcus* spp., and (e) other colors = Gram-positive microorganism other than *Streptococcus uberis*; *Streptococcus agalactiae*; *Streptococcus dysgalactiae*; *Enterococcus* spp. or *Lactococcus* spp. (other Gram-positive microorganism).
- Section 2—Gram-negative: (a) purple = *E. coli*; (b) metallic blue = *Klebsiella* spp., *Enterobacter* spp., or *Serratia* spp.; (c) yellow = *Pseudomonas* spp.; (d) white and dry = yeast and *Prototheca* spp., and (e) other colors = Gram-negative microorganism other than *E. coli; Klebsiella* spp.; *Enterobacter* spp.; *Serratia* spp. or *Pseudomonas* spp. (other Gram-negative microorganism).
- Section 3—(a) pink = *Staphylococcus aureus*; (b) other colors = other bacteria from *Staphylococcus* spp genus other than *Staphylococcus aureus* (non-aureus staphylococci).

## Development of the AI-based mobile application

### Convolutional neural networks model: inputs, training and the validation procedure

Machine learning methods based on supervised learning were used to create an automatic classifier based on a convolutional neural networks model to make an automated diagnosis of mastitis-causing pathogens growth in chromogenic culture media (Smartcolor 2). The image database contained images of Petri dishes that presented growth of at least one microorganism colony isolated from milk samples from mastitic cows. All images were captured at the farms using OnFarm OFC system and were registered using the OnFarmApp.

Before the training procedure, all the images were labeled by a specialist (PhD veterinarian specializing in microbiology, with six years of experience in microbiological identification by the chromogenic culture media triplate used in the study) with bounding boxes indicating on images the object's region (microorganism’s colonies) with their respective object classes (positive diagnosis)^31^. The labeled database with target information (classes for positive diagnosis) is the procedure in a supervised learning approach that indicates what the model should learn using the data from the dataset in the training process^32^.

### Experimental set-up and evaluation metrics

An experimental set using the Petri dishes images was adopted to create the classifier of the automatic mastitis-causing pathogen diagnosis. For this, we used an open-source model for object detection in images named YoloV5 version “M”^33^. The main model’s features used in this study were: model size = 42.2 megabytes; trainable parameters = 20,875,359; image size = 640 X 640 and depth = 169. The main hyperparameters used in the training procedure were: optimization algorithm = stochastic gradient descent; batch size = 32; momentum = 0.937; weight decay = 0.0005 and learning rate = 0.01.

To the training process we adopted the weights and a trained model using the “transfer learning” approach in our application. In this procedure, the model (YoloV5m) was trained using the Common Objects in Context dataset (COCO) composed of over 330 thousand images, around 1.5 million instances of objects to detect 80 different types of objects in images^34^. The transfer learning technique uses a trained model as a starting point (mode = l’s weights) to train a new model for a new context using new images, for new objects, new classes and especially, using a smaller training dataset^35^. Our application was implemented using the programming language Python 3.8^36^ combined with PyTorch 1.8^37^ as back-end. To evaluate the classifier model, the Diagnostic Accuracy Measures was adopted^38^, which was composed by four parameters: True Positives (TP), True Negatives (TN), False Positives (FP) and False Negatives (FN), that enabled the analyses of the Accuracy (Acc), Sensitivity (Se), Specificity (Sp), Positive Predictive Value (PPV) and Negative Predictive Value (NPV).

To develop the classifier, we utilized 1,550 images randomly selected from the OnFarmApp database, which contained around 450,000 mastitis case images recorded by FPU during standard OFC procedures. All images underwent encryption of farm and cow information prior to selection to safeguard the privacy of the farms involved. The dataset was divided randomly into two subsets, representing 80% and 20% from the dataset. In the first subset, the total of 1,240 images were selected for the training process and in the second, 310 images were selected for the validation process. To evaluate entire database in the training process, we adopted the k-fold cross-validation method^39,40^. In our experiment a fivefold cross-validation procedure was used, randomly separating the dataset five times with 80% of images for training the classifiers and 20% of images for testing ensuring the best model at the end of the training process. After the training procedure, the machine learning model has been deployed a Rest service with a HTTP protocol developed in Flask Python in an EC2 service hosted in an Amazon Web Service (AWS). Then, a mobile and web application was built (Rumi, OnFarm, Piracicaba, Brazil), which was integrated with the machine learning algorithms services using an Application Program Interface (API). The service worked uploading the digital image in the service and getting back the result from the Rumi AI-based application service.

## Evaluation of the reliability of the AI-based application

The database for training and validation, composed of 1,550 images, was used to ensure the best model at the end of the training process. Meanwhile, the test data set, which was not used in the training procedure, was a sample of unknown data for the trained model. The test dataset contained 684 images, including 476 from plates with bacteria previously identified by Matrix-Assisted Laser Desorption Ionization—Time-of-Flight Mass Spectrometry (MALDI-TOF MS), as described by Granja et al. (2021). The remaining 208 images were randomly selected from the OnFarmApp image database, and identified by the trained specialist.

## Trial 1 Diagnostic accuracy of Rumi and a trained specialist for the identification of CM causing pathogens in chromogenic culture media images, using MALDI-TOF MS as the reference (Fig. 2)

**Figure 2:**
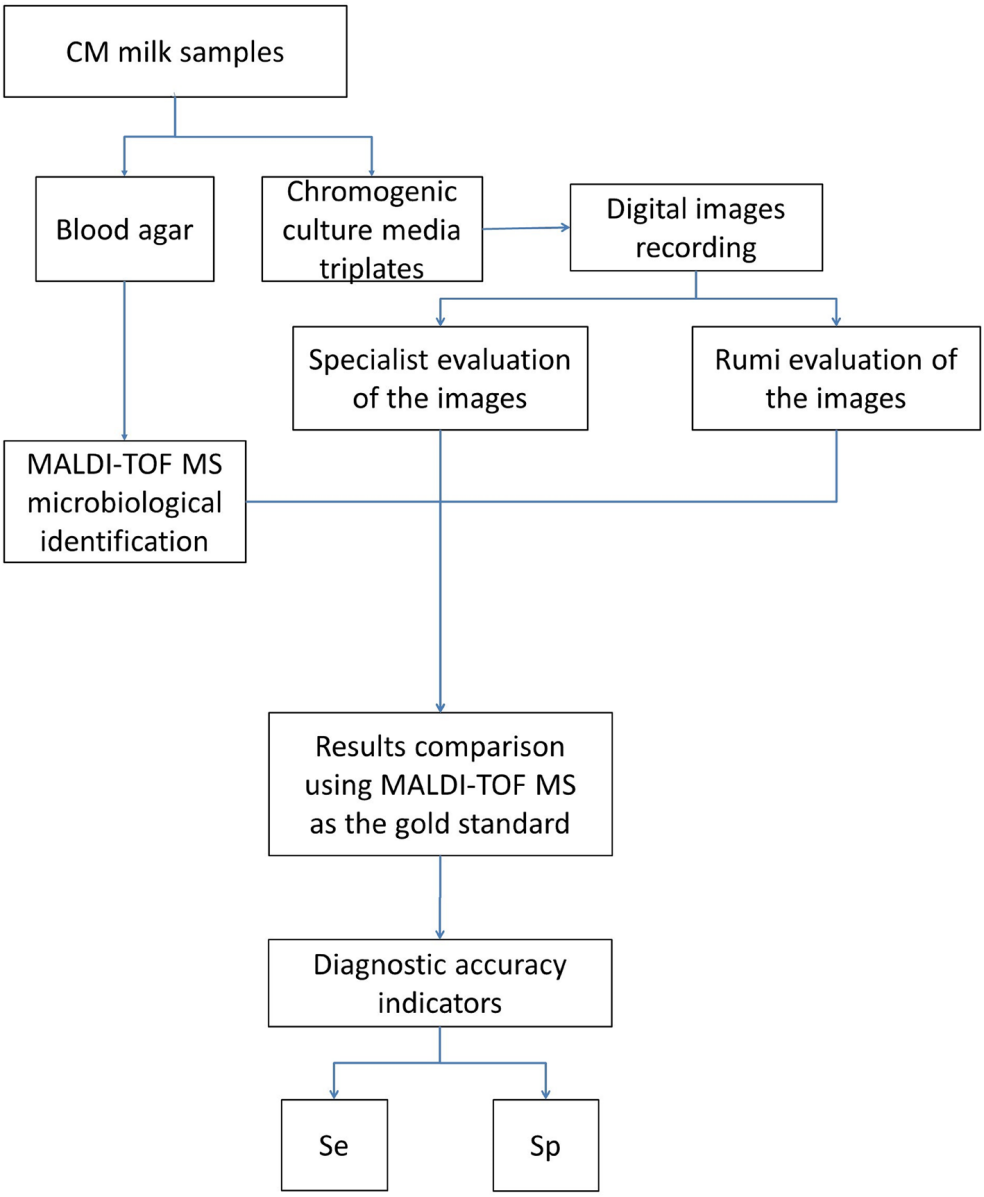
Trial 1 Flowchart—Estimation of the Sensitivity (Se) and Specificity (Sp) of the artificial intelligence-based application (Rumi; OnFarm, Piracicaba, São Paulo, Brazil) and the trained specialist to identify pathogens growing on chromogenic culture media on farms. MALDI-TOF MS was considered as the gold standard.

### Objective

Evaluate the diagnostic accuracy of Rumi using MALDI-TOF MS as the gold standard, and compare its results with the accuracy of a specialist with six years of experience in microbiological identification using the chromogenic culture media triplate Smartcolor 2.

#### Images, data collection and diagnostic accuracy under laboratory conditions

A total of 476 images of SmartColor 2 triplate with colony growth from CM milk samples, originating from a previous study^4^ were used in Trial 1. These images were generated from 476 CM cases, from 441 cows of 25 farms located in two states of Brazil (São Paulo and Minas Gerais), selected as a non-probabilistic convenience sampling. The number of samples was chosen in accordance with the comparable literature^41,42^. Briefly, all CM milk samples were sent from the farm to the laboratory frozen at −20°C and then, in laboratory conditions, milk samples were inoculated, with a sterile swab, simultaneously in SmartColor 2 and in blood agar. After 24 h (for SmartColor 2) and 24 to 48 hours (for blood agar) of incubation at 37°C, the plates were inspected and all microbiological colonies grown in SmartColor 2 and in blood agar were submitted to species identification using MALDI-TOF MS. Concurrently, the SmartColor 2 plates were photographed for further evaluation.

All images were classified by the following two methods: (a) a trained specialist (not involved in the previous study) and (b) Rumi. The specialist had access to the digital images and recorded a presumptive diagnosis based on colonies color patterns and growth on selective media, following manufacturer’s recommendations. Rumi’s readings were carried out by uploading and processing digital images using the Web OnFarmApp.

#### Diagnostic performance indicators

The diagnostic accuracy (Se, Sp and accuracy), for the microbiological identification of the specialist and Rumi, were estimated using the MALDI-TOF MS microbiological identification results as gold standard. The recorded number of colonies of each isolate was considered to classify a sample as positive. In our criteria, all samples with the isolation of less than three colonies (with the exception of *Staphylococcus aureus*), were classified as negative for that particular species. Images of plates displaying the isolation of two different morphologies of colonies were classified as mixed culture, and considered positive for the two species in the analysis. Contaminated samples (defined as the presence of three or more morphologically-distinct colonies in the same sample) were not included in the analysis. Pathogens with a frequency of isolation lower than 10 were grouped as “other pathogens”.

For each pathogen group evaluated, samples were considered TP when microbiological growth was observed, and the visual presumptive identification of chromogenic media coincided with the identification in MALDI-TOF MS for isolates in blood-agar. A sample was considered TN when no microbiological growth with color pattern associated with this specie was observed in chromogenic media and no identification of the species was done by MALDI-TOF MS in blood-agar isolates of the same sample. A sample was considered FP when there was an isolation of microorganism with different identification result between the chromogenic culture media and MALDI-TOF MS identification of blood-agar isolates of the same sample. Finally, a sample was considered FN when no bacterial growth with color pattern associated with this specie was observed in chromogenic media, but a microbiological identification of the pathogen was made in MALDI-TOF MS for blood-agar isolates of the same sample.

The diagnostic performance indicators were calculated using the software R Studio (version 4.1.3). Using the recorded results of TP, TN, FP and TN. A confusion matrix was created using bdpv package^43^, and the results were used to generate the Se and Sp, as well as the Wald confidence intervals (0.95 confidence limits). McNemar’s Exact tests were used for comparing sensitivities and specificities between the specialist and Rumi for each pathogen group.

## Trial 2 Diagnostic accuracy of Rumi and farm personnel users for the identification of mastitis-causing pathogens in chromogenic culture media plates (Fig. 3)

**Figure 3:**
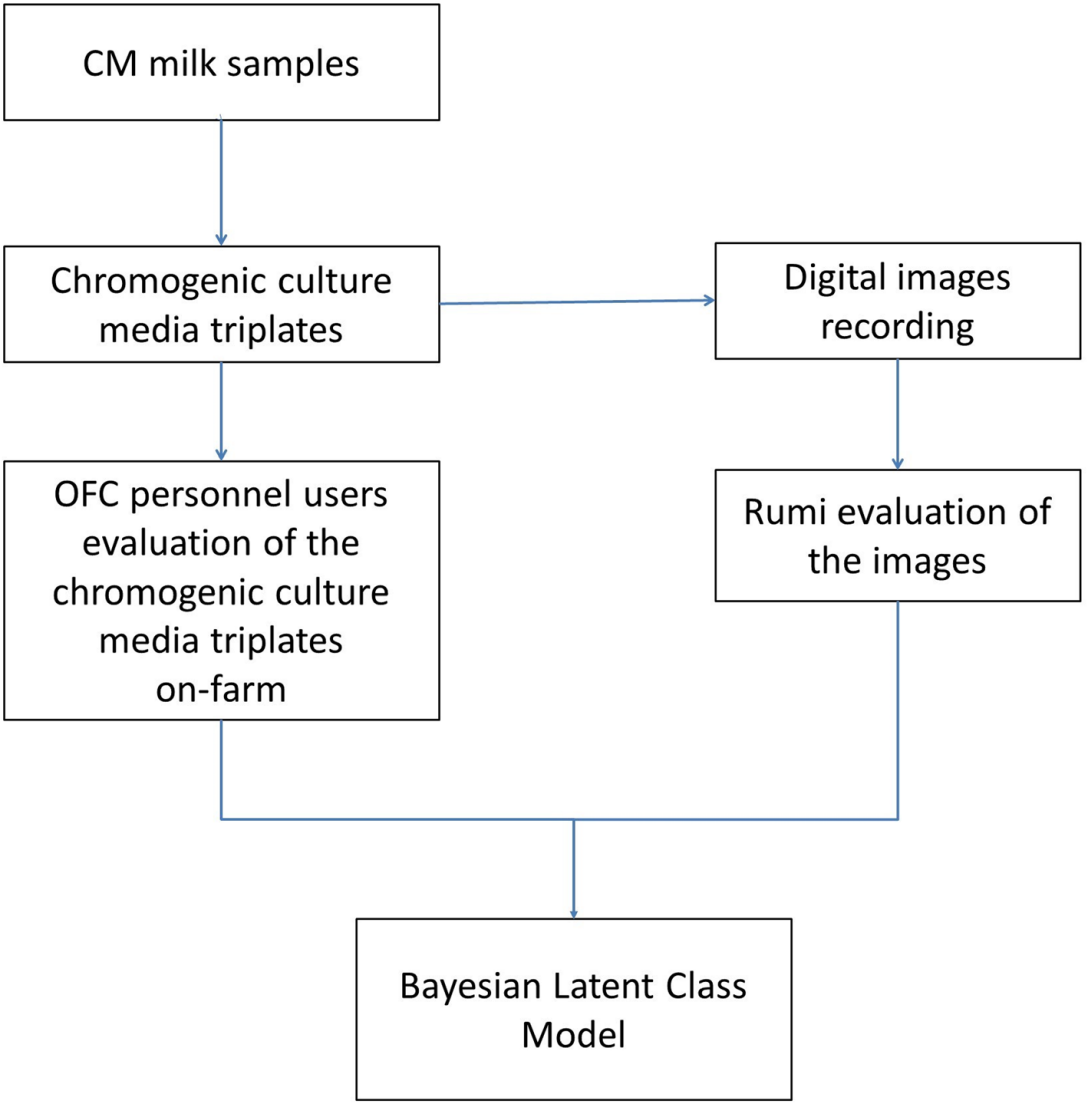
Trial 2 Flowchart—A comparison of Sensitivity (Se) and Specificity (Sp) between the artificial intelligence-based application (Rumi; OnFarm, Piracicaba, São Paulo, Brazil) and farm personnel users for the identification of clinical mastitis pathogens in images from chromogenic culture media plates.

### Objective

Compare the diagnostic accuracy of Rumi and FPU to estimate potential gains of using Rumi for the interpretation of OFC results.

#### Images and Data Selection

For this trial, we selected 208 images, originating from 150 Brazilian dairy farms located in three Brazilian states (Minas Gerais, São Paulo and Paraná). Images were randomly selected from a pool of eligible images meeting the following criteria: (1) Image captured using the Onfarm’s reader support located at the top of the SmartLab incubator; (2) Image of a plate placed in the correct position at the reader support and (3) Image without environmental interferences (e.g., camera flashes, objects in front of the plate). No minimum specification was required for the devices or image quality. The images, as well as the FPU’s presumptive microbiological identification, were previously recorded in OnFarmApp. Additionally, images were also identified using the Rumi.

Milk samples were considered culture-positive if 3 or more colonies with the color patterns defined for the species were present. The only exception was *Staphylococcus aureus,* in which the growth of a single colony was considered positive. Mixed samples were defined as the presence of 2 distinct species in sufficient numbers on the same sample. Plates were considered contaminated when > 2 different morphology of colonies were present. Mixed culture plates were considered positive for both groups of pathogens whereas contaminated samples were not included in the analyses.

#### Bayesian Latent Class Model

Since two distinct tests (Rumi and FPU reading) were used, and none of the two could be regarded as a gold standard for bacterial identification, we opted for a Bayesian approach to estimate the differences in sensitivity and specificity between Rumi and FPU. This difference served as a proxy for assessing the potential on-farm gains in diagnostic accuracy through automated plate reading. Trial 2 contained no missing data or indeterminate results.

To accomplish this, we developed a set of Bayesian latent class models following the STARD-BLCM guidelines (Table S1). These models were tailored for each group of pathogens, taking into account the utilization of the two tests within a particular population^44^. The latent variable in this instance was a positive culture result on blood agar for a species or group of species, as identified by MALDI-TOF. The two tests were considered independent from one another. A multinomial distribution was used to represent all possible 4 outcome combinations, as follows:

$$y_{observed} = multinomial\left( {P_{{observed\left[ {1:4} \right]}} , n} \right)$$

$$P_{observed\left[ 1 \right]} = P_{population} \times \left( {Se_{Rumi} \times Se_{FPU} } \right) + \left( {1 - P_{population} } \right) \times \left( {\left( {1 - Sp_{Rumi} } \right) \times \left( {1 - Sp_{FPU} } \right)} \right)$$

$$P_{observed\left[ 2 \right]} = P_{population} \times \left( {Se_{Rumi} \times (1 - Se_{FPU} )} \right) + \left( {1 - P_{population} } \right) \times \left( {\left( {1 - Sp_{Rumi} } \right) \times Sp_{FPU} } \right)$$

$$P_{observed\left[ 3 \right]} = P_{population} \times \left( {\left( {1 - Se_{Rumi} } \right) \times Se_{FPU} } \right) + \left( {1 - P_{population} } \right) \times \left( {Sp_{Rumi} \times \left( {1 - Sp_{FPU} } \right)} \right)$$

$$P_{observed\left[ 4 \right]} = P_{population} \times \left( {\left( {1 - Se_{Rumi} } \right) \times (1 - Se_{FPU} )} \right) + \left( {1 - P_{population} } \right) \times \left( {Sp_{Rumi} \times Sp_{FPU} } \right)$$

where y_observed_ is a vector that denotes the number of observed results after n trials that fall in each possible combination according to the diagnostic test results, assumed to follow a multinomial distribution with cell probability P_observed_. P_population_ represents the true prevalence of each group of pathogens. P_observed_ [1] to [4] represent the different probabilities of samples being classified as test positive or negative in each diagnostic test according to the true pathogen prevalence. Se_FPU_, Se_Rumi_, Sp_FPU_ and Sp_Rumi_, represent the sensitivities and specificities of the FPU and Rumi, respectively. A sample code is available as supplementary material (Supplementary Text 1).

Prior information on the Se and Sp of Rumi as well as true prevalence priors were incorporated into the Bayesian latent class models (Table 5). These were chosen according to the Trial 1 results. Non-informative priors were used for the Se and Sp of FPU. Priors were determined using the *betaExpert* function in R^45^. Distributions were not truncated and could attain any value in the parameter space. We carried a set of sensitivity analysis considering alternative prevalence priors, as well as weaker diagnostic priors for Rumi with identical modes.

**Table 5:** Prior distributions used in final Bayesian Latent Class models and their interpretation.

Pathogen	Parameter	Distribution	Mode	95% certainty that true value
*Streptococcus agalactiae/Streptococcus dysgalactiae*	Prevalence	(1.93, 20.30)	0.05	< 20%
	Sensitivity	(15.43, 7.73)	0.68	> 50%
	Specificity	(9.20, 1.17)	0.98	> 70%
*Streptococcus uberis*	Prevalence	(1.75, 19.06)	0.04	< 20%
	Sensitivity	(26.18, 5.72)	0.84	> 70%
	Specificity	(10.29, 1.40)	0.96	> 70%
*Enterococcus* spp.	Prevalence	(1.49, 23.66)	0.02	< 20%
	Sensitivity	(1.34, 2.36)	0.20	> 5%
	Specificity	(9.47, 1.22)	0.97	> 70%
*Klebsiella* spp*./Enterobacter* spp*./Serratia* spp*.*	Prevalence	(2.59, 24.66)	0.06	< 20%
	Sensitivity	(9.84, 1.30)	0.97	> 70%
	Specificity	(8.65, 1.05)	0.99	> 70%
*Escherichia coli*	Prevalence	(3.30, 29.06)	0.08	< 20%
	Sensitivity	(16.44, 2.93)	0.89	> 70%
	Specificity	(8.57, 1.03)	1.00	> 70%
*Staphylococcus aureus*	Prevalence	(3.16, 28.24)	0.07	< 20%
	Sensitivity	(10.02, 4.12)	0.74	> 50%
	Specificity	(9.03, 1.13)	0.98	> 70%
*Non-aureus* staphylococci	Prevalence	(2.86, 16.18)	0.11	< 30%
	Sensitivity	(10.78, 4.60)	0.73	> 50%
	Specificity	(9.16, 1.16)	0.98	> 70%

A Markov chain Monte Carlo approach using Gibbs sampling was performed with 4 chains in parallel with a total of 400,000 iterations using the *runjags* package in R^46^. Visual inspection of the chains, effective sample sizes, and autocorrelation plots were used as measures of efficacy. An effective sample size of at least 10,000 was required for all parameters. The *step()* function was used to estimate the probability of the Se and Sp of Rumi being greater than those of the FPU for the identification of each group of pathogens. Statistical significance was considered at the 5% level. These analyses were carried out in R.

### Supplementary Information

Supplementary Information.

## Data Availability

The datasets used and/or analyzed during the current study available from the corresponding author on reasonable request.

## References

[1] Pol M, Ruegg PL (2007). Treatment practices and quantification of antimicrobial drug usage in conventional and organic dairy farms in Wisconsin. J. Dairy Sci..

[2] Suojala L, Kaartinen L, Pyörälä S (2013). Treatment for bovine *Escherichia coli* mastitis—An evidence-based approach. J. Vet. Pharmacol. Ther..

[3] de Jong E (2023). Invited review: Selective treatment of clinical mastitis in dairy cattle. J. Dairy Sci..

[4] Granja BM, Fidelis CE, Garcia BLN, dos Santos MV (2021). Evaluation of chromogenic culture media for rapid identification of microorganisms isolated from cows with clinical and subclinical mastitis. J. Dairy Sci..

[5] Roberson JR (2003). Establishing treatment protocols for clinical mastitis. Vet. Clin. N. Am. Food Anim. Pract..

[6] Lago A, Godden SM (2018). Use of rapid culture systems to guide clinical mastitis treatment decisions. Vet. Clin. N. Am. Food Anim. Pract..

[7] de Jong E (2023). Selective treatment of nonsevere clinical mastitis does not adversely affect cure, somatic cell count, milk yield, recurrence, or culling: A systematic review and meta-analysis. J. Dairy Sci..

[8] Sipka A (2021). Short communication: Comparative performance of 3 on-farm culture systems for detection of mastitis pathogens interpreted by trained and untrained observers. J. Dairy Sci..

[9] Glasson J (2017). Multicenter evaluation of an image analysis device (APAS): Comparison between digital image and traditional plate reading using urine cultures. Ann. Lab. Med..

[10] Spampinato C, Palazzo S, Giordano D, Aldinucci M, Leonardi R (2017). Deep learning for automated skeletal bone age assessment in X-ray images. Med. Image Anal..

[11] Porto LF (2019). Automatic cephalometric landmarks detection on frontal faces: An approach based on supervised learning techniques. Digit. Investig..

[12] Porto LF (2020). Estimating sex and age from a face: A forensic approach using machine learning based on photo-anthropometric indexes of the Brazilian population. Int. J. Legal Med..

[13] Khan SU, Islam N, Jan Z, Ud Din I, Rodrigues JJPC (2019). A novel deep learning based framework for the detection and classification of breast cancer using transfer learning. Pattern Recognit. Lett..

[14] Franco A (2022). Diagnostic performance of convolutional neural networks for dental sexual dimorphism. Sci. Rep..

[15] Shaily, T. & Kala, S. Bacterial image classification using convolutional neural networks. In *Bacterial Image Classification Using Convolutional Neural Networks* 1–6 (2020).

[16] Abade A, Porto LF, Ferreira PA, Vidal FDB (2021). NemaNet: A convolutional neural network model for identification of nematodes soybean crop in Brazil. Biosyst. Eng..

[17] Gammel N (2021). Comparison of an automated plate assessment system (APAS independence) and artificial intelligence (AI) to manual plate *Staphylococcus aureus* CHROMagar surveillance cultures. J. Clin. Microbiol..

[18] Faron ML, Buchan BW, Samra H, Ledeboera NA (2020). Evaluation of WASPLab software to automatically read chromid CPS elite agar for reporting of urine cultures. J. Clin. Microbiol..

[19] Baker J, Timm K, Faron M, Ledeboer N, Culbreath K (2021). Digital image analysis for the detection of group B *Streptococcus* from ChromID strepto B medium using phenomatrix algorithms. J. Clin. Microbiol..

[20] Van TT, Mata K, Bard JD (2019). Automated detection of streptococcus pyogenes pharyngitis by use of colorex strep a CHROMagar and WASPLab artificial intelligence chromogenic detection module software. J. Clin. Microbiol..

[21] Freu G (2023). Association between mastitis occurrence in dairy cows and bedding characteristics of compost-bedded pack barns. Pathogens.

[22] Ferreira JC (2018). Comparative analysis of four commercial on-farm culture methods to identify bacteria associated with clinical mastitis in dairy cattle. PLoS One.

[23] Fuenzalida MJ, Ruegg PL (2019). Negatively controlled, randomized clinical trial to evaluate intramammary treatment of nonsevere, gram-negative clinical mastitis. J. Dairy Sci..

[24] Keefe G (2012). Update on control of *Staphylococcus aureus* and *Streptococcus agalactiae* for management of mastitis. VFP.

[25] Garcia BLN, Fidelis CE, Freu G, Granja BDM, dos Santos MV (2021). Evaluation of chromogenic culture media for rapid identification of gram-positive bacteria causing mastitis. Front. Vet. Sci..

[26] Obaidat MM, Bani Salman AE, Roess AA (2018). High prevalence and antimicrobial resistance of mecA *Staphylococcus aureus* in dairy cattle, sheep, and goat bulk tank milk in Jordan. Trop. Anim. Health Prod..

[27] Tomazi T (2014). Identification of coagulase-negative staphylococci from bovine intramammary infection by matrix-assisted laser desorption ionization-time of flight mass spectrometry. J. Clin. Microbiol..

[28] Braga PAC (2018). Rapid identification of bovine mastitis pathogens by MALDI-TOF Mass Spectrometry. Pesquisa Veterinaria Brasileira.

[29] NMC (2017). Laboratory Handbook on Bovine Mastitis.

[30] Griffioen K, Velthuis AGJ, Koop G, Lam TJGM (2021). Effects of a mastitis treatment strategy with or without on-farm testing. J. Dairy Sci..

[31] Liu L (2020). Deep Learning for generic object detection: A survey. Int. J. Comput. Vis..

[32] Sathya R, Abraham A (2013). Comparison of supervised and unsupervised learning algorithms for pattern classification. Int. J. Adv. Res. Artif. Intell..

[33] Jocher, G. *et al.* ultralytics/yolov5: v6.0—YOLOv5n ‘Nano’ models, Roboflow integration, TensorFlow export, OpenCV DNN support. Preprint at 10.5281/ZENODO.5563715 (2021).

[34] Lin, T. *et al.* Microsoft COCO : Common objects in context. In *European Conference on Computer Vision* 740–755 (2014).

[35] Torrey, L. & Shavlik, J. Transfer Learning. In *Handbook of Research on Machine Learning Applications and Trends* 242–264 (IGI Global, 2010). 10.4018/978-1-60566-766-9.ch011.

[36] Van Rossum, G. & Drake, F. L. *Python 3 Reference Manual. Scotts: CreateSpace.* (2011).

[37] Paszke, A. *et al.* PyTorch: An imperative style, high-performance deep learning library. In *33rd Conference on Neural Information Processing Systems* 8026–8037 (2019).

[38] Eusebi P (2013). Diagnostic accuracy measures. Cerebrovasc. Dis..

[39] Kohavi, R. A study of cross-validation and bootstrap for accuracy estimation and model selection. In *International Joint Conference on Artificial Intelligence* 1137–1145 (1995).

[40] Hastie, T., Tibshirani, R. & Friedman, J. *The Elements of Statistical Learning Data Mining, Inference, and Prediction*. (2009).

[41] Ganda EK, Bisinotto RS, Decter DH, Bicalho RC (2016). Evaluation of an on-farm culture system (Accumast) for fast identification of milk pathogens associated with clinical mastitis in dairy cows. PLoS One.

[42] Lago A, Godden SM, Bey R, Ruegg PL, Leslie K (2011). The selective treatment of clinical mastitis based on on-farm culture results: I. Effects on antibiotic use, milk withholding time, and short-term clinical and bacteriological outcomes. J. Dairy Sci..

[43] Package, T. & Inference, T. Package ‘bdpv’. Preprint at (2022).

[44] Branscum AJ, Gardner IA, Johnson WO (2005). Estimation of diagnostic-test sensitivity and specificity through Bayesian modeling. Prev. Vet. Med..

[45] Devleesschauwer, B., Torgerson, P. R., Charlier, J. & Levecke, B. Package ‘prevalence’. Preprint at 10.5167/uzh-89061 (2013).

[46] Denwood MJ (2016). runjags: An R package providing interface utilities, model templates, parallel computing methods and additional distributions for MCMC models in JAGS. J. Stat. Softw..

