# Supplementary material for: Accuracy of an AI-based automated plate reading mobile application for the identification of clinical mastitis-causing pathogens in chromogenic culture media
Source: Sci Rep. 2024 Jan 12;14:1208. doi: 10.1038/s41598-023-50296-w (PMC10786835; doi:10.1038/s41598-023-50296-w)
Supplement: Supplementary file 1 — Supplementary Information. [file 41598_2023_50296_MOESM1_ESM.docx]

**Supplementary text.** JAGS Model string for 2 diagnostic tests used for detecting *Streptococcus agalactiae* or *Streptococcus dysgalactiae* in culture plates.

model {

x[1:4] ~ dmulti(p[1:4], n)

p[1] <- Prev*(Se_ml*Se_user) + (1-Prev)*((1-Sp_ml)*(1-Sp_user))

p[2] <- Prev*(Se_ml*(1-Se_user)) + (1-Prev)*((1-Sp_ml)*Sp_user)

p[3] <- Prev*((1-Se_ml)*Se_user) + (1-Prev)*(Sp_ml*(1-Sp_user))

p[4] <- Prev*((1-Se_ml)*(1-Se_user)) + (1-Prev)*(Sp_ml*Sp_user)

Prev ~ dbeta(1.930743, 20.30281)

Se_user ~ dbeta(1, 1)

Sp_user ~ dbeta(1, 1)

Se_ml ~ dbeta(15.42801,7.733634)

Sp_ml ~ dbeta(9.19926, 1.165625)

ml_wins_se <- step(Se_ml - Se_user)

ml_wins_sp <- step(Sp_ml - Sp_user)

}

Supplementary tables

|  |  | **Rumi** |  | **Specialist** | |
| --- | --- | --- | --- | --- | --- |
| Pathogen | MALDI-TOF MS |  |  | Positive | Negative |
| *Streptococcus agalactiae* /  *Streptococcus dysgalactiae* | + | Positive |  | 2 | 5 |
|  |  | Negative |  | 1 | 14 |
|  | - | Positive |  | 439 | 6 |
|  |  | Negative |  | 1 | 8 |
| *Streptococcus uberis* | + | Positive |  | 2 | 1 |
|  |  | Negative |  | 0 | 16 |
|  | - | Positive |  | 434 | 4 |
|  |  | Negative |  | 12 | 7 |
| *Enterococcus* spp. | + | Positive |  | 4 | 4 |
|  |  | Negative |  | 0 | 2 |
|  | - | Positive |  | 451 | 3 |
|  |  | Negative |  | 0 | 12 |
| *Klebsiella* spp. /  *Enterobacter* spp. / *Serratia* spp. | + | Positive |  | 1 | 0 |
|  |  | Negative |  | 1 | 28 |
|  | - | Positive |  | 443 | 0 |
|  |  | Negative |  | 1 | 2 |
| *Escherichia coli* | + | Positive |  | 1 | 3 |
|  |  | Negative |  | 0 | 32 |
|  | - | Positive |  | 437 | 1 |
|  |  | Negative |  | 0 | 2 |
| *Staphylococcus aureus* | + | Positive |  | 4 | 5 |
|  |  | Negative |  | 2 | 24 |
|  | - | Positive |  | 433 | 1 |
|  |  | Negative |  | 4 | 3 |
| *Non-aureus* staphylococci | + | Positive |  | 3 | 11 |
|  |  | Negative |  | 0 | 38 |
|  | - | Positive |  | 415 | 1 |
|  |  | Negative |  | 5 | 3 |

**Table 1** Cross-tabulated results of the visual identification of mastitis-causing pathogens from clinical mastitis samples (n=476) in chromogenic culture media triplates (SmartColor 2 - On Farm. Brazil) made by a trained specialist and by an artificial intelligence-based application (Rumi; OnFarm. Piracicaba. São Paulo. Brazil), according to the MALDI-TOF MS status of the sample.

|  | **Rumi** |  | **Farm user** | |
| --- | --- | --- | --- | --- |
| Pathogen |  |  | Positive | Negative |
| *Streptococcus agalactiae* /  *Streptococcus dysgalactiae* | Positive |  | 22 | 4 |
|  | Negative |  | 11 | 171 |
| *Streptococcus uberis* | Positive |  | 18 | 6 |
|  | Negative |  | 10 | 174 |
| *Enterococcus* spp. | Positive |  | 6 | 8 |
|  | Negative |  | 7 | 187 |
| *Klebsiella* spp. /  *Enterobacter* spp. / *Serratia* spp. | Positive |  | 9 | 0 |
|  | Negative |  | 1 | 198 |
| *Escherichia coli* | Positive |  | 15 | 2 |
|  | Negative |  | 3 | 188 |
| *Staphylococcus aureus* | Positive |  | 15 | 4 |
|  | Negative |  | 15 | 174 |
| *Non-aureus* staphylococci | Positive |  | 39 | 14 |
|  | Negative |  | 23 | 132 |

**Table 2** Cross tabulated results of the visual identification of mastitis-causing pathogens from clinical mastitis milk samples (n=208) in chromogenic culture media triplates (SmartColor 2 - On Farm. Brazil) made by farm personnel users (FPU) and by an artificial intelligence-based application (Rumi; OnFarm. Piracicaba. São Paulo. Brazil).

| Species | Prevalence value | Distribution | Comparison | Probability |
| --- | --- | --- | --- | --- |
| *Streptococcus agalactiae* / | 0.05 | (1.93, 20.30) | Se Rumi > Se FPU | 0.06 |
| *Streptococcus dysgalactiae* |  |  | Sp Rumi > Sp FPU | 0.67 |
|  | 0.10 | (5.62, 42.57) | Se Rumi > Se FPU | 0.06 |
|  |  |  | Sp Rumi > Sp FPU | 0.67 |
|  | 0.15 | (5.04, 23.90) | Se Rumi > Se FPU | 0.06 |
|  |  |  | Sp Rumi > Sp FPU | 0.63 |
| *Streptococcus uberis* | 0.04 | (1.75, 19.06) | Se Rumi > Se FPU | 0.37 |
|  |  |  | Sp Rumi > Sp FPU | 0.72 |
|  | 0.09 | (4.46, 35.98) | Se Rumi > Se FPU | 0.38 |
|  |  |  | Sp Rumi > Sp FPU | 0.72 |
|  | 0.14 | (4.33, 21.48) | Se Rumi > Se FPU | 0.39 |
|  |  |  | Sp Rumi > Sp FPU | 0.71 |
| *Enterococcus* spp. | 0.02 | (1.49, 23.66) | Se Rumi > Se FPU | 0.33 |
|  |  |  | Sp Rumi > Sp FPU | 0.33 |
|  | 0.07 | (2.96, 26.96) | Se Rumi > Se FPU | 0.36 |
|  |  |  | Sp Rumi > Sp FPU | 0.34 |
|  | 0.12 | (3.28, 17.74) | Se Rumi > Se FPU | 0.39 |
|  |  |  | Sp Rumi > Sp FPU | 0.36 |
| *Klebsiella* spp. / | 0.06 | (2.59, 24.66) | Se Rumi > Se FPU | 0.50 |
| *Enterobacter* spp. / *Serratia* spp. |  |  | Sp Rumi > Sp FPU | 0.65 |
|  | 0.11 | (2.89, 16.3) | Se Rumi > Se FPU | 0.50 |
|  |  |  | Sp Rumi > Sp FPU | 0.64 |
|  | 0.16 | (2.96, 11.3) | Se Rumi > Se FPU | 0.50 |
|  |  |  | Sp Rumi > Sp FPU | 0.64 |
| *Escherichia coli* | 0.08 | (3.30, 29.06) | Se Rumi > Se FPU | 0.41 |
|  |  |  | Sp Rumi > Sp FPU | 0.59 |
|  | 0.13 | (3.76, 19.45) | Se Rumi > Se FPU | 0.42 |
|  |  |  | Sp Rumi > Sp FPU | 0.58 |
|  | 0.18 | (3.60, 12.84) | Se Rumi > Se FPU | 0.42 |
|  |  |  | Sp Rumi > Sp FPU | 0.58 |
| *Staphylococcus aureus* | 0.07 | (3.16, 28.24) | Se Rumi > Se FPU | 0.18 |
|  |  |  | Sp Rumi > Sp FPU | 0.92 |
|  | 0.12 | (3.28, 17.74) | Se Rumi > Se FPU | 0.16 |
|  |  |  | Sp Rumi > Sp FPU | 0.89 |
|  | 0.17 | (3.26, 12.02) | Se Rumi > Se FPU | 0.16 |
|  |  |  | Sp Rumi > Sp FPU | 0.88 |
| *Non-aureus* staphylococci | 0.11 | (2.86, 16.18) | Se Rumi > Se FPU | 0.22 |
|  |  |  | Sp Rumi > Sp FPU | 0.69 |
|  | 0.16 | (2.96, 11.30) | Se Rumi > Se FPU | 0.22 |
|  |  |  | Sp Rumi > Sp FPU | 0.67 |
|  | 0.21 | (2.83, 7.88) | Se Rumi > Se FPU | 0.22 |
|  |  |  | Sp Rumi > Sp FPU | 0.65 |

**Table 3** Sensitivity analysis of Bayesian Latent Class models using different prior information for true prevalence values for the different pathogens causing mastitis. In each model, we compared the probability of the artificial intelligence-based algorithm (Rumi) test characteristic being higher than its counterpart in the second test (farm personnel user [FPU]). Se = sensitivity; Sp = Specificity.

| Species | Rumi Characteristic | Distribution | 95% certainty that true value | Comparison | Probability |
| --- | --- | --- | --- | --- | --- |
| *Streptococcus agalactiae* / | Se | (3.80, 2.31) | > 30% | Se Rumi > Se FPU | 0.16 |
| *Streptococcus dysgalactiae* | |  |  | Sp Rumi > Sp FPU | 0.74 |
|  | Sp | (4.49, 1.07) | > 50% | Se Rumi > Se FPU | 0.06 |
|  |  |  |  | Sp Rumi > Sp FPU | 0.67 |
| *Streptococcus uberis* | Se | (6.42, 2.02) | > 50% | Se Rumi > Se FPU | 0.36 |
|  |  |  |  | Sp Rumi > Sp FPU | 0.68 |
|  | Sp | (4.69, 1.16) | > 50% | Se Rumi > Se FPU | 0.39 |
|  |  |  |  | Sp Rumi > Sp FPU | 0.74 |
| *Enterococcus* spp. | Se | (5.15, 17.62) | > 10% | Se Rumi > Se FPU | 0.07 |
|  |  |  |  | Sp Rumi > Sp FPU | 0.17 |
|  | Sp | (4.54, 1.09) | > 50% | Se Rumi > Se FPU | 0.33 |
|  |  |  |  | Sp Rumi > Sp FPU | 0.33 |
| *Klebsiella* spp. / | Se | (4.61, 1.12) | > 50% | Se Rumi > Se FPU | 0.44 |
| *Enterobacter* spp. /*Serratia* spp. | |  |  | Sp Rumi > Sp FPU | 0,63 |
|  | Sp | (4.37, 1.02) | > 50% | Se Rumi > Se FPU | 0.50 |
|  |  |  |  | Sp Rumi > Sp FPU | 0.65 |
| *Escherichia coli* | Se | (5.55, 1.57) | > 50% | Se Rumi > Se FPU | 0.42 |
|  |  |  |  | Sp Rumi > Sp FPU | 0.59 |
|  | Sp | (4.36, 1.02) | > 50% | Se Rumi > Se FPU | 0.41 |
|  |  |  |  | Sp Rumi > Sp FPU | 0.58 |
| *Staphylococcus aureus* | Se | (3.34, 1.81) | > 30% | Se Rumi > Se FPU | 0.22 |
|  |  |  |  | Sp Rumi > Sp FPU | 0.90 |
|  | Sp | (4.45, 1.06) | > 50% | Se Rumi > Se FPU | 0.18 |
|  |  |  |  | Sp Rumi > Sp FPU | 0.92 |
| *Non-aureus* staphylococci | Se | (3.42, 1.89) | > 30% | Se Rumi > Se FPU | 0.34 |
|  |  |  |  | Sp Rumi > Sp FPU | 0.75 |
|  | Sp | (4.48, 1.07) | > 50% | Se Rumi > Se FPU | 0.21 |
|  |  |  |  | Sp Rumi > Sp FPU | 0.66 |

**Table 4** Sensitivity analysis of Bayesian Latent Class models fit using different prior information for the artificial intelligence-based algorithm (Rumi) test characteristics. In each model, we compared the probability of the characteristic being higher for Rumi than for farm personnel users (FPU). Se = sensitivity; Sp = Specificity.

|  | **Section & Topic** | **No** | **Item** | **Reported on page #** |
| --- | --- | --- | --- | --- |
|  |  |  |  |  |
|  | **TITLE OR ABSTRACT** |  |  |  |
|  |  | **1** | Identification as a study of diagnostic accuracy, using at least one measure of accuracy (such as sensitivity, specificity, predictive values, or AUC) **and Bayesian latent class models** | 1 |
|  | **ABSTRACT** |  |  |  |
|  |  | **2** | Structured summary of study design, methods, results, and conclusions  (for specific guidance, see STARD for Abstracts) | 1 |
|  | **INTRODUCTION** |  |  |  |
|  |  | **3** | Scientific and clinical background, including the intended use and clinical role of the **tests under evaluation** | 2 |
|  |  | **4** | Study objectives and hypotheses, **such as estimation of diagnostic accuracy of the tests for a defined purpose through BLCM** | 2 |
|  | **METHODS** |  |  |  |
|  | *Study design* | **5** | Whether data collection was planned before the **tests** were performed (prospective study) or after (retrospective study) | 7 and 8 |
|  | *Participants* | **6** | Eligibility criteria **and description of the source population** | 7 and 8 |
|  |  | **7** | On what basis potentially eligible participants were identified  (such as symptoms, results from previous tests, inclusion in registry) | 7 and 8 |
|  |  | **8** | Where and when potentially eligible participants were identified (setting, location and dates) | 7 and 8 |
|  |  | **9** | Whether participants formed a consecutive, random or convenience series | 7 and 8 |
|  | *Test methods* | **10** | **Description of the tests under evaluation**, in sufficient detail to allow replication, **and/or cite references** | 5 and 6 |
|  |  | **11** | Rationale for choosing the **tests under evaluation in relation to their purpose** | 2 |
|  |  | **12** | Definition of and rationale for test positivity cut-offs or result categories of **the tests under evaluation**, distinguishing pre-specified from exploratory | 7 and 8 |
|  |  | **13** | Whether clinical information was available to the performers or readers of **the tests under evaluation** | 7 and 8 |
|  | *Analysis* | **14a** | **BLCM model** for estimating measures of diagnostic accuracy | 8 and 9 |
|  |  | **14b** | **Definition and rationale of prior information and sensitivity analysis** | 8 |
|  |  | **15** | How indeterminate results **of the tests under evaluation** were handled | 8 |
|  |  | **16** | How missing data **of the tests under evaluation** were handled | 8 |
|  |  | **17** | Any analyses of variability in diagnostic accuracy, distinguishing pre-specified from exploratory | 8 |
|  |  | **18** | Intended sample size and how it was determined | 7 |
|  | **RESULTS** |  |  |  |
|  | *Participants* | **19** | Flow of participants, using a diagram | 13 and 14 |
|  |  | **20** | Baseline demographic and clinical characteristics of participants | 2 and 3 |
|  |  | **21** | **Not applicable: the distribution of the targeted conditions is unknown, hence the use of BLCM** | NA |
|  |  | **22** | Time interval and any clinical interventions between **the tests under evaluation** | NA |
|  | *Test results* | **23** | Cross tabulation of the **tests’ results (or for continuous tests results their distribution by infection stage)** | 3 |
|  |  | **24** | Estimates of diagnostic accuracy **under alternative prior specification** and their precision (such as 95% **credible/probability intervals**) | 3 |
|  |  | **25** | Any adverse events from performing **the tests under evaluation** | NA |
|  | **DISCUSSION** |  |  |  |
|  |  | **26** | Study limitations, including sources of potential bias, statistical uncertainty, and generalisability | 4 and 5 |
|  |  | **27** | Implications for practice, including the intended use and clinical role of **the tests under evaluation in relevant settings (clinical, research, surveillance etc.)** | 4 and 5 |
|  | **OTHER INFORMATION** |  |  |  |
|  |  | **28** | Registration number and name of registry | NA |
|  |  | **29** | Where the full study protocol can be accessed | NA |
|  |  | **30** | Sources of funding and other support; role of funders | 12 |
|  |  |  |  |  |

STARD – BLCM

STARD-BLCM stands for “Standards for the Reporting of Diagnostic accuracy studies that use Bayesian Latent Class Models” and is a modification of the STARD statement (which was recently updated to STARD2015). STARD-BLCM aims to facilitate improved quality of reporting for diagnostic accuracy studies that use Bayesian latent class models in the absence of a reference standard. The proposed modifications are relevant to both Bayesian and frequentist estimation methods but the focus is on the former.

More information for STARD (STARD2015) can be found at: [http://www.equator-network.org/reporting-guidelines/stard](http://www.equator-network.org/reporting-guidelines/stard/)

More information for STARD-BLCM can be found at: [http://www.equator-network.org/reporting-guidelines/stard-blcm](http://www.equator-network.org/reporting-guidelines/stard-blcm/)
